# Are skin senescence and immunosenescence linked within individuals?

**DOI:** 10.1111/acel.12956

**Published:** 2019-05-06

**Authors:** Mariëtte E. C. Waaijer, David Goldeck, David A. Gunn, Diana van Heemst, Rudi G. J. Westendorp, Graham Pawelec, Andrea B. Maier

**Affiliations:** ^1^ Department of Internal Medicine, Section Gerontology and Geriatrics Leiden University Medical Center Leiden The Netherlands; ^2^ Department of Internal Medicine II, Centre for Medical Research University of Tübingen Tübingen Germany; ^3^ Unilever Discover Sharnbrook, Bedfordshire UK; ^4^ Department of Public health and Center of Healthy Aging University of Copenhagen Copenhagen Denmark; ^5^ Health Sciences North Research Institute Sudbury Ontario Canada; ^6^ Division of Cancer Studies, Faculty of Life Sciences and Medicine King's College London London UK; ^7^ Department of Human Movement Sciences, @AgeAmsterdam, Amsterdam Movement Sciences Vrije Universiteit Amsterdam The Netherlands; ^8^ Department of Medicine and Aged Care, @AgeMelbourne, Royal Melbourne Hospital University of Melbourne Melbourne Victoria Australia

**Keywords:** cellular senescence, human, immunosenescence, skin aging

## Abstract

With advancing age, many organs exhibit functional deterioration. The age‐associated accumulation of senescent cells is believed to represent one factor contributing to this phenomenon. While senescent cells are found in several different organ systems, it is not known whether they arise independently in each organ system or whether their prevalence within an individual reflects that individual's intrinsic aging process. To address this question, we studied senescence in two different organ systems in humans, namely skin and T cells in 80 middle‐aged and older individuals from the Leiden Longevity Study. Epidermal p16INK4a positivity was associated with neither CD4^+^ nor CD8^+^ T‐cell immunosenescence phenotype composites (i.e., end‐stage differentiated/senescent T cells, including CD45RA^+^CCR7^‐^CD28^‐^CD27^‐^CD57^+^KLRG1^+^ T cells). Dermal p16INK4a positivity was significantly associated with the CD4^+^, but not with the CD8^+^ immunosenescence composite. We therefore conclude that there is limited evidence for a link between skin senescence and immunosenescence within individuals.

## 1 INTRODUCTION

One of the processes hypothesized to underlie age‐related functional decline in organ systems throughout the body is cellular senescence (Lopez‐Otin, Blasco, Partridge, Serrano, & Kroemer, 2013). This state of cell cycle arrest is believed to be irreversible under physiological conditions. In human skin, the prevalence of senescent cells is higher in aged individuals than in young (Ressler et al., 2006). Previously, we observed that the number of skin cells positive for the cell cycle control protein p16INK4a, commonly accepted to be a marker of cellular senescence, was lower in offspring from long‐living families and linked to cardiovascular disease (Waaijer et al., 2012). This suggests that skin aging occurs at a different pace in different individuals.

While the skin constitutes an important barrier, the immune system represents another organ system essential for protection against harmful environmental exposures throughout life. With age, several changes occur in the adaptive immune system, broadly termed immunosenescence. The number of naïve T cells (characterized by their expression of costimulatory receptors such as CD28 and CD27) decreases with age and differentiated memory, and effector T‐cell numbers increase (Arnold, Wolf, Brunner, Herndler‐Brandstetter, & Grubeck‐Loebenstein, 2011). This represents an essential characteristic of adaptive immunity and is more marked in older people because of their previous exposures to pathogens. Other receptors such as CD57 and killer lectin‐like receptor G1 (KLRG1), which deliver negative signals to T cells, are also more frequently found on T cells of older individuals and are commonly considered to mark senescence (Brenchley et al., 2003; Voehringer, Koschella, & Pircher, 2002).

To study whether senescence occurs at the same pace in different organ systems, we studied 80 participants (aged 45–81 years) of the Leiden Longevity Study (LLS), assessing whether the amount of p16INK4a‐positive cells in skin correlates with the amount of putatively immunosenescent T cells in blood. The mean age was 61 years, 48.8% were female, and half were seropositive for cytomegalovirus (CMV) (Table S1). The distribution of the markers studied is given in Table S2.

**Figure 1:**
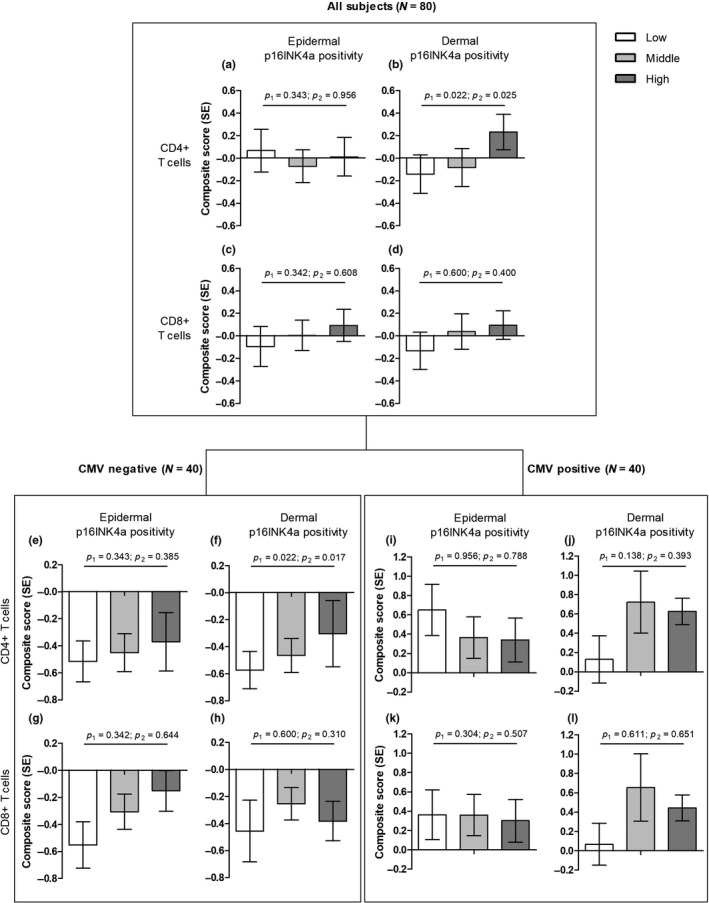
The association between T‐cell immunosenescence composite scores and p16INK4a positivity in human skin. The means of the T‐cell immunosenescence composite score in the crude model per p16INK4a tertile are shown. P_1_ is the p‐value for the crude model, p_2_ for model 2 (adjustment for age, gender, long‐lived family membership, and sunbed use). All subjects: (a) epidermal p16INK4a positivity and CD4^+^ immunosenescence composite; (b) epidermal p16INK4a positivity and CD8^+^ immunosenescence composite; (c) dermal p16INK4a positivity and CD4^+^ immunosenescence composite; (d) dermal p16INK4a positivity and CD8^+^ immunosenescence composite. (e–h) show the same analyses for CMV‐negative subjects, (i–j) for CMV‐positive subjects

The main results are given in Figure 1 and Table 1. Epidermal p16INK4a positivity was not associated with either the CD4^+^ or CD8^+^ immunosenescence composite score (all models: *p* > 0.05). Dermal p16INK4a positivity was significantly associated with the CD4^+^ immunosenescence composite score both in the crude model and after adjustment for age, gender, long‐lived family membership, and sunbed use (model 1, *p* = 0.022; model 2, *p* = 0.025). Dermal p16INK4a positivity was not associated with the CD8^+^ composite score (all models: *p* > 0.05). When stratified for CMV serostatus, the positive association between dermal p16INK4a positivity with the CD4+ composite score was only present in CMV‐negative subjects (model 1, *p* = 0.022; model 2, *p* = 0.017). In Table S3, the results for the three single components of the composite score are shown. The association is mainly driven by cells with the CD45RA^‐^CCR7^‐^CD28^‐^CD27^‐^ and KLRG1^+^ phenotype. To test whether (epi)dermal p16INK4a positivity was linked to immune responses of CD4^+^ or CD8^+^ T cells, we studied this in a subset of subjects (*N* = 66). We did not find any significant association between in vitro flu‐peptide stimulation responses and (epi)dermal p16INK4A positivity (Table S4).

**Table 1:** The association between T‐cell immunosenescence composite scores and absolute numbers of p16INK4a positivity in human skin

	Epidermal p16INK4a positivity		Dermal p16INK4a positivity	
	*β* (*SE*)	*p*‐value	*β* (*SE*)	*p*‐value
All subjects (*N* = 80)				
CD4^+^ composite				
Model 1	0.029 (0.030)	0.343	0.086 (0.036)	0.022
Model 2	0.002 (0.032)	0.956	0.093 (0.041)	0.025
CD8^+^ composite				
Model 1	0.027 (0.028)	0.342	0.019 (0.036)	0.600
Model 2	0.014 (0.028)	0.608	0.031 (0.036)	0.400
CMV‐negative (*N* = 40)				
CD4^+^ composite				
Model 1	0.029 (0.030)	0.343	0.086 (0.036)	0.022
Model 2	0.028 (0.031)	0.385	0.093 (0.037)	0.017
CD8^+^ composite				
Model 1	0.027 (0.028)	0.342	0.019 (0.036)	0.600
Model 2	0.014 (0.030)	0.644	0.039 (0.038)	0.310
CMV‐positive (*N* = 40)				
CD4^+^ composite				
Model 1	0.003 (0.046)	0.956	0.097 (0.064)	0.138
Model 2	−0.013 (0.046)	0.788	0.058 (0.067)	0.393
CD8^+^ composite				
Model 1	0.045 (0.043)	0.304	0.032 (0.063)	0.611
Model 2	0.027 (0.041)	0.507	−0.027 (0.059)	0.651

Note

Linear regression, data are given as β (standard error).

Model 1: crude model. Model 2: as model 1 plus adjustment for age, gender, and sunbed use. Composite scores: Z‐scores of CD45RA− CCR7− CD28− CD27−, CD57+, and KLRG1+, divided by 3.

We present unique data on senescence markers in two different organ systems in humans: the skin and the immune system. We hypothesized that if both organ systems age at a similarly pace within an individual, we would find associations between skin senescence and immunosenescence. Both organs show senescent features to a higher degree with advancing age. Additional to exterior and histological changes in the skin at older age, higher numbers of senescent cells have been found (Ressler et al., 2006). These senescent cells might contribute to the aging process, as they are associated with age‐related pathology (Bhat et al., 2012; Verzola et al., 2008). Clearance of senescent cells delayed and ameliorated age‐related pathologies in mice (Baker et al., 2011). Senescence of immune cells with advancing age has also been described (Solana et al., 2012). Some of these immunosenescence features, especially those that form the “Immune Risk Profile” (Ferguson, Wikby, Maxson, Olsson, & Johansson, 1995), are linked to mortality. Senescence has been mostly described in different organs separately but in some studies, senescence levels were reported for different tissues, showing a diversity in fold changes in old vs. young mice across tissues (Baker et al., 2011; Krishnamurthy et al., 2004).

In our study, dermal p16INK4a positivity was positively associated with CD4^+^ T‐cell immunosenescence markers, showing an intra‐individual link. The association remained after adjustment for age, gender, and sunbed use showing that this link is not solely driven by these factors. This association was only present in CMV‐negative subjects, indicating that the intra‐individual link might be obscured by increased immunosenescence due to CMV infection. This is in line with another study where an association between KLRG‐1 and aging phenotypes (increased adipose tissue or decreased muscle mass) in HIV‐infected patients was found (Tavenier et al., 2015). However, similar to CMV infection, HIV infection appears to strongly affect CD8+ T‐cell differentiation and maturation, because there was no association between CD8+ differentiation and aging phenotypes. Thus, one reason for the limited associations found between skin and immunosenescence might be that other factors accelerate immunosenescence but not skin senescence, obscuring the intra‐individual association. Indeed, our results were not consistent for both skin compartments and T‐cell subsets because epidermal p16INK4 positivity was not associated with immunosenescence and dermal p16INK4a positivity was not associated with CD8^+^ T‐cell immunosenescence. Another explanation for this might be the weak intra‐individual association between epidermal (melanocyte) and dermal (fibroblast) p16INK4a positivity (Spearman correlation coefficient 0.069, *p* = 0.541).

We have used unique data on skin senescence and immunosenescence within the same middle‐aged to older individuals, who have previously been shown to differ in their biological age (Derhovanessian et al., 2010; Waaijer et al., 2016, 2012). The number of participants was limited to those individuals who had available data on both skin senescence and immunosenescence (original groups were randomly selected from the cohort). A limitation is that we used immunosenescence composite scores based on markers that were previously linked to age of the donor (Koch et al., 2008) and to replicative senescence (Larbi & Fulop, 2014). However, differences in percentages of subsets between young and old individuals might not reflect actual functionality of the immune system per se as these immune cells with low replication capacity have, for example, higher cytokine production (Larbi & Fulop, 2014). Also, we do not have data on the immune function of our participants, such as vaccination responses or incidence of infections. Although a lower proportion of CMV seropositive elderly donors have a CD4^+^ T‐cell response to flu‐peptide stimulation (Derhovanessian et al., 2014), we did not find any association between in vitro flu‐peptide stimulation responses and p16INK4a positivity in the skin (subset *N* = 66). We used only one marker of senescence in the skin biopsies; inclusion of another marker could have improved the identification of the senescence state.

In conclusion, we find evidence for a link between dermal p16INK4a positivity and CD4^+^ T‐cell immunosenescence markers but no compelling link overall between skin senescence and immunosenescence. Further work would be needed to replicate and expand these findings.

## CONFLICT OF INTEREST

None declared.
