# Supplementary material for: Are skin senescence and immunosenescence linked within individuals?
Source: Aging Cell. 2019 May 6;18(4):e12956. doi: 10.1111/acel.12956 (PMC6612632; doi:10.1111/acel.12956)
Supplement: Supplementary file 1 [file ACEL-18-e12956-s001.docx]

**Supplementary material**

**Author contributions**

MECW, DG, GP and ABM devised the outline of this research paper (research questions, analyses); DG performed the immune cell flow cytometry; MECW did the statistical analyses and wrote the paper; RGJW designed the LLS; DAG led the p16 staining in skin biopsies; all authors critically read and revised the manuscript.

**Funding**

This work was supported by the European Union-funded Network of Excellence LifeSpan (FP6 036894), DFG-PA 361/14-1, DFG-SFB685-B4, and DFG-PA 361/11-1, the Innovation Oriented Research Program on Genomics (SenterNovem; IGE01014 and IGE5007), the Centre for Medical Systems Biology, The Netherlands Genomics Initiative/Netherlands Organization for Scientific Research (05040202 and 050-060-810), Unilever PLC and IDEAL.

**Conflict of interest**DAG is a Unilever employee. Although no products were tested, this work could potentially promote the use of anti-ageing products and lead to financial gain for Unilever.

**Experimental procedures**

*Study design and participants*

In the Leiden Longevity Study (LLS) factors contributing to familial longevity are studied; the study design was previously published (Schoenmaker et al., 2006). Approval by the Medical Ethical Committee of the Leiden University Medical Center was obtained. Demographic and medical characteristics were obtained for these participants from their general practitioners or through questionnaires. For this present study data on both p16INK4a positivity in skin biopsies and T-cell immunosenescence markers of 40 offspring of nonagenarian siblings and their 40 partners was available.

*Skin biopsies – p16INK4a immunohistochemistry*

As described previously (Waaijer et al., 2012), 4 mm skin biopsies were taken from the upper inner arm and fixed in formalin, paraffin embedded and cut in 4 µm sections. The sections were stained for p16INK4a by using the E6H4 antibody (CINtec Histology Kit; MTM Laboratories). Positive staining cells were counted separately in the epidermis and dermis, with dermal staining targeted to fibroblast like cells (isolated cells in proximity to collagen fibres) and corrected for the measurement area (per mm of the epidermal-dermal junction, and per 1mm^2^ of the dermis respectively).

*Serum – CRP, CMV, and T-cell characterisation*

High sensitive C-reactive protein (CRP) levels were measured in serum with dual count solid phase no boil assay (Diagnostic Products, Los Angeles, CA). CMV serostatus was measured by ELISA (CMV-IgG-ELISA PKS assay, Medac GmbH, Wedel, Germany) per manufacturer’s instructions.

Although p16ink4a expression in human T-cells has been linked to chronological aging and specifically accelerated aging in HIV+ patients (Liu et al., 2009; Nelson et al., 2012), in other studies p16ink4a expression was not informative for replicative senescence in selected long-term cultured monoclonal CD4+ T-cells (Hyland et al., 2001). Here, we therefore obtained peripheral blood mononuclear cells (PBMCs) from participants and flow cytometry was performed for surface molecules thought to be associated with immunosenescence, as described in earlier work (Derhovanessian et al., 2010). Briefly, staining was carried out in PFEA buffer (PBS, 2% FCS, 2 mM EDTA, and 0.01% azide). PBMCs were thawed, rested and treated with human Ig (Gamunex, Bayer, Leverkusen, Germany) and ethidium monoazide (EMA) (Invitrogen, Karlsruhe, Germany) on ice for 10 minutes to block Fc-receptors and label nonviable cells. First, PBMCs were stained with anti–KLRG-1 primary antibody (a kind gift of Prof. H. Pircher, Freiburg, Germany) on ice for 20 minutes, then stained with Pacific Orange-conjugated goat anti-mouse IgG (Invitrogen) on ice for 20 minutes. To block nonspecific binding to anti-mouse secondary antibody, mouse serum (Chemicon/Millipore, Schwalbach, Germany) was added for 15 minutes. Directly conjugated monoclonal antibodies were then added: CD3-PE (Calltag; Invitrogen), CD4-PerCP, CD8-allophycocyanin-Cy7, CCR7-PE-Cy7 (BD Biosciences, Heidelberg, Germany), CD27-allophycocyanin, CD45RA-Pacific Blue, CD28-Alexa Fluor 700 (BioLegend, San Diego, CA) and CD57-FITC (Immunotools, Freiburg, Germany). After 20 minutes incubation on ice the cells were washed, and immediately analysed on an LSR II cytometer with FACSDiva software (BD Biosciences). EMA-positive dead cells were excluded from the analysis. Characterisation of T-cell subsets (CD3^+^) was done according to (Koch et al., 2008) and also previously described within the LLS (Derhovanessian et al., 2010). The gating strategy can also be found in this paper. The methods for in vitro flu-peptide stimulation were previously detailed in (Derhovanessian et al., 2014). A participant was considered a responder if the percentage of cells producing IFNγ, TNF or IL-2 upon flu-peptide stimulation was ≥2-fold that of unstimulated cells.

*Composites*

Based on a previous study (Koch et al., 2008) we selected differentiation/senescence marker-based (CD27, CD28, CD57, KLRG1) subsets of T-cells that were associated with chronological age. Per individual, the percentages of these subsets were combined in a composite score to reflect overall immunosenescence of T-cells. This was done by adding Z-scores of subset percentages and dividing by the total number of subsets (3) in the composite score. If percentages of subsets were not normally distributed they were first naturally log transformed before computing the Z-score. These composites were calculated separately for the CD4^+^ and CD8^+^ subsets. One composite score consisted of the Z scores of the fraction of ln CD45RA^-^ CCR7^-^ CD28^-^ CD27^-^, ln CD57^+^ and ln KLRG1^+^ within CD4^+^ T-cells (termed CD4^+^ immunosenescence composite score). The other composite score consisted of the Z scores of the fraction of ln CD45RA^-^ CCR7^-^ CD28^-^ CD27^-^, CD57^+^ and KLRG1^+^ within CD8^+^ T-cells (termed CD8^+^ immunosenescence composite score). A higher composite score reflects a higher number of positive senescence markers.

*Statistics*

All analyses were carried out using IBM SPSS Statistics 20 software; the Figure was made with GraphPad Prism 5.01 software. The data was analysed by using linear regression (except for the flu-peptide stimulation data). The associations between epidermal/dermal p16INK4a positivity (independent variables) and immunosenescence composite scores (dependent variables) were analysed according to 2 models. Model 1 was the crude model; model 2 included adjustments for age, gender, membership of a long-lived family and sunbed use. For data visualisation we divided the epidermal and dermal p16 counts into tertiles. For epidermal p16INK4a positivity these tertiles were: low <0.17 (N=26), middle 0.17-1.05 (N=28) and high >1.05 cells (N=26) staining positive for p16INK4a per mm length of the epidermal–dermal junction. For dermal p16INK4a positivity: low <0.88 (N=26), middle 0.88-2.45 (N=28), high >2.45 (N=26) cells staining positive for p16INK4a per 1 mm2 dermis. The data on flu-peptide stimulation and epidermal/dermal p16INK4a positivity were tested by using logistic regression. Model 1 was the crude model, model 2 included adjustment for age, gender, sunbed use and long-lived family membership, and model 3 was as model 2 plus adjustment for CMV serostatus.

**References**

Derhovanessian, E., Maier, A. B., Beck, R., Jahn, G., Hahnel, K., Slagboom, P. E., de Craen, A. J., Westendorp, R. G., & Pawelec, G. (2010). Hallmark features of immunosenescence are absent in familial longevity. *J.Immunol., 185*(8), 4618-4624.

Derhovanessian, E., Maier, A. B., Hahnel, K., McElhaney, J. E., Slagboom, E. P., & Pawelec, G. (2014). Latent infection with cytomegalovirus is associated with poor memory CD4 responses to influenza A core proteins in the elderly. *J Immunol, 193*(7), 3624-3631.

Hyland, P., Barnett, C., Pawelec, G., & Barnett, Y. (2001). Age-related accumulation of oxidative DNA damage and alterations in levels of p16(INK4a/CDKN2a), p21(WAF1/CIP1/SDI1) and p27(KIP1) in human CD4+ T cell clones in vitro. *Mech Ageing Dev, 122*(11), 1151-1167.

Koch, S., Larbi, A., Derhovanessian, E., Ozcelik, D., Naumova, E., & Pawelec, G. (2008). Multiparameter flow cytometric analysis of CD4 and CD8 T cell subsets in young and old people. *Immun.Ageing, 5*, 6.

Liu, Y., Sanoff, H. K., Cho, H., Burd, C. E., Torrice, C., Ibrahim, J. G., Thomas, N. E., & Sharpless, N. E. (2009). Expression of p16(INK4a) in peripheral blood T-cells is a biomarker of human aging. *Aging Cell, 8*(4), 439-448.

Nelson, J. A., Krishnamurthy, J., Menezes, P., Liu, Y., Hudgens, M. G., Sharpless, N. E., & Eron, J. J., Jr. (2012). Expression of p16(INK4a) as a biomarker of T-cell aging in HIV-infected patients prior to and during antiretroviral therapy. *Aging Cell, 11*(5), 916-918.

Schoenmaker, M., de Craen, A. J., de Meijer, P. H., Beekman, M., Blauw, G. J., Slagboom, P. E., & Westendorp, R. G. (2006). Evidence of genetic enrichment for exceptional survival using a family approach: the Leiden Longevity Study. *Eur.J.Hum.Genet., 14*(1), 79-84.

Waaijer, M. E., Parish, W. E., Strongitharm, B. H., van, H. D., Slagboom, P. E., de Craen, A. J., Sedivy, J. M., Westendorp, R. G., Gunn, D. A., & Maier, A. B. (2012). The number of p16INK4a positive cells in human skin reflects biological age. *Aging Cell, 11*(4), 722-725.

| **Supplementary table 1. Characteristics of study participants.** | | | | | | |
| --- | --- | --- | --- | --- | --- | --- |
|  |  |  | CMV serostatus | |  | Subset flu-peptide stimulation (N=66) |
|  | All (N=80) |  | Negative (N=40) | Positive (N=40) |  |  |
| Female, no. (%) | 39 (48.8) |  | 19 (47.5) | 20 (50.0) |  | 32 (48.5) |
| Age, years | 61.1 (56.9-68.4) |  | 58.8 (55.7-66.9) | 64.1 (58.6-69.1) |  | 60.8 (56.8-67.6) |
| Offspring of nonagenarian siblings, no. (%) | 40 (50.0) |  | 22 (55.0) | 18 (45.0) |  | 35 (53.0) |
| Body mass index, kg/m^2^ | 26.0 (23.8-27.8) |  | 25.3 (23.1-27.4) | 26.5 (25.0-28.0) |  | 26.0 (23.8-27.6) |
| Comorbidities, no (%) |  |  |  |  |  |  |
| Cerebrovascular accident | 2 (2.9) |  | 0 (0.0) | 2 (6.1) |  | 1 (1.8) |
| Chronic obstructive pulmonary disease | 2 (2.9) |  | 0 (0.0) | 2 (6.1) |  | 2 (3.6) |
| Diabetes mellitus | 2 (2.9) |  | 1 (2.9) | 1 (3.0) |  | 2 (3.6) |
| Hypertension | 15 (22.4) |  | 5 (12.5) | 10 (31.3) |  | 13 (23.6) |
| Malignancy | 3 (4.4) |  | 1 (2.9) | 2 (6.1) |  | 3 (5.4) |
| Myocardial infarction | 1 (1.3) |  | 0 (0.0) | 1 (3.0) |  | 1 (1.8) |
| Rheumatoid arthritis | 0 (0.0) |  | 0 (0.0) | 0 (0.0) |  | 0 (0.0) |
| Smoking, current and former, no (%) | 50 (67.6) |  | 25 (65.8) | 25 (69.4) |  | 39 (65.0) |
| Sun bed use | 1 (1-2) |  | 1 (1-2) | 1 (1-2) |  | 1 (1-2) |
| hsCRP, mg/L | 1.36 (0.63-2.45) |  | 0.96 (0.40-2.20) | 1.59 (0.75-3.06) |  | 1.32 (0.63-2.36) |
| CMV serostatus |  |  |  |  |  |  |
| Positive, no (%) | 40 (50.0) |  | n/a | n/a |  | 30 (45.5) |
| Responder flu-peptide |  |  |  |  |  |  |
| CD4^+^ | n/a |  | n/a | n/a |  | 51 (77.3) |
| CD8^+^ | n/a |  | n/a | n/a |  | 36 (54.5) |
| Continuous data is given as median (interquartile range). Missing data on N=12 for comorbidities, N=6 for smoking and hsCRP, N=3 for BMI. n/a: not applicable. Scale sunbed use: 1 (never) to 3 (≥6x per year). | | | | | | |

| **Supplementary table 2. Distribution of p16INK4a positive skin cells and T-cell immunosenescence markers.** | | |
| --- | --- | --- |
|  | Median (IQR) | |
| **P16INK4a positivity** |  |  |
| Epidermis, no. per mm of epidermal-dermal junction | 0.52 (0.13-1.52) | |
| Dermis, no. per 1mm^2^ | 1.42 (0.43-3.04) | |
|  |  |  |
| **Individual components immunosenescence, %** | **CD4^+^** | **CD8^+^** |
| CD45RA^-^ CCR7^-^ CD28^-^ CD27^-^ | 0.20 (0.08-0.67) | 1.86 (0.89-3.86) |
| CD57^+^ | 0.92 (0.49-3.20) | 24.6 (12.0-35.3) |
| KLRG1^+^ | 2.73 (1.13-5.10) | 42.5 (30.6-58.4) |
| No.: number. IQR: interquartile range. The distribution of the individual components within the composite score is given. | | |

| **Supplementary table 3. The association between the different T-cell immunosenescence markers within the composite score and p16INK4a positivity in human skin** | | | | | | | | | | | | | | |  |  |  |
| --- | --- | --- | --- | --- | --- | --- | --- | --- | --- | --- | --- | --- | --- | --- | --- | --- | --- |
|  | **All subjects (N=80)** | | | | |  | **CMV negative (N=80)** | | | | |  | **CMV positive (N=80)** | | | | |
|  | **Epidermal p16INK4a positivity** | |  | **Dermal p16INK4a positivity** | |  | **Epidermal p16INK4a positivity** | |  | **Dermal p16INK4a positivity** | |  | **Epidermal p16INK4a positivity** | |  | **Dermal p16INK4a positivity** | |
|  | β (SE) | P-value |  | β (SE) | P-value |  | β (SE) | P-value |  | β (SE) | P-value |  | β (SE) | P-value |  | β (SE) | P-value |
| **CD4^+^** |  |  |  |  |  |  |  |  |  |  |  |  |  |  |  |  |  |
| **CD45RA^-^CCR7**  **^-^CD28^-^CD27^-^** |  |  |  |  |  |  |  |  |  |  |  |  |  |  |  |  |  |
| Model 1 | 0.036 (0.036) | 0.323 |  | 0.012 (0.047) | 0.020 |  | 0.026 (0.039) | 0.513 |  | 0.144 (0.043) | 0.002 |  | 0.037 (0.054) | 0.495 |  | 0.095 (0.077) | 0.227 |
| Model 2 | 0.021 (0.037) | 0.574 |  | 0.120 (0.047) | 0.013 |  | 0.025 (0.042) | 0.558 |  | 0.157 (0.047) | 0.002 |  | 0.038 (0.055) | 0.494 |  | 0.040 (0.080) | 0.616 |
| **CD57^+^** |  |  |  |  |  |  |  |  |  |  |  |  |  |  |  |  |  |
| Model 1 | 0.020 (0.036) | 0.585 |  | 0.060 (0.048) | 0.216 |  | 0.017 (0.034) | 0.624 |  | 0.020 (0.043) | 0.644 |  | 0.011 (0.053) | 0.842 |  | 0.153 (0.073) | 0.042 |
| Model 2 | -0.005 (0.036) | 0.881 |  | 0.078 (0.047) | 0.104 |  | 0.013 (0.033) | 0.701 |  | 0.016 (0.043) | 0.703 |  | -0.007 (0.053) | 0.900 |  | 0.109 (0.075) | 0.155 |
| **KLRG1^+^** |  |  |  |  |  |  |  |  |  |  |  |  |  |  |  |  |  |
| Model 1 | 0.008 (0.036) | 0.823 |  | 0.064 (0.048) | 0.192 |  | 0.044 (0.040) | 0.273 |  | 0.093 (0.049) | 0.063 |  | -0.040 (0.056) | 0.475 |  | 0.044 (0.081) | 0.587 |
| Model 2 | -0.010 (0.038) | 0.790 |  | 0.081 (0.049) | 0.106 |  | 0.045 (0.043) | 0.304 |  | 0.105 (0.052) | 0.053 |  | -0.069 (0.059) | 0.248 |  | 0.023 (0.087) | 0.791 |
|  |  |  |  |  |  |  |  |  |  |  |  |  |  |  |  |  |  |
| **CD8^+^** |  |  |  |  |  |  |  |  |  |  |  |  |  |  |  |  |  |
| **CD45RA^-^CCR7^-^**  **CD28^-^CD27^-^** |  |  |  |  |  |  |  |  |  |  |  |  |  |  |  |  |  |
| Model 1 | 0.052 (0.036) | 0.150 |  | 0.079 (0.048) | 0.106 |  | 0.042 (0.038) | 0.275 |  | 0.088 (0.047) | 0.068 |  | 0.058 (0.061) | 0.345 |  | 0.079 (0.089) | 0.380 |
| Model 2 | 0.042 (0.037) | 0.257 |  | 0.080 (0.048) | 0.102 |  | 0.034 (0.042) | 0.421 |  | 0.108 (0.051) | 0.040 |  | 0.071 (0.062) | 0.258 |  | 0.007 (0.091) | 0.937 |
| **CD57^+^** |  |  |  |  |  |  |  |  |  |  |  |  |  |  |  |  |  |
| Model 1 | 0.029 (0.036) | 0.425 |  | -0.029 (0.049) | 0.552 |  | 0.005 (0.036) | 0.886 |  | -0.032 (0.045) | 0.478 |  | 0.044 (0.054) | 0.419 |  | 0.006 (0.079) | 0.942 |
| Model 2 | -0.006 (0.036) | 0.865 |  | -0.005 (0.048) | 0.918 |  | -0.013 (0.036) | 0.724 |  | -0.023 (0.046) | 0.614 |  | 0.015 (0.053) | 0.781 |  | -0.050 (0.076) | 0.513 |
| **KLRG1^+^** |  |  |  |  |  |  |  |  |  |  |  |  |  |  |  |  |  |
| Model 1 | 0.037 (0.036) | 0.316 |  | -0.003 (0.049) | 0.958 |  | 0.034 (0.041) | 0.413 |  | 0.001 (0.052) | 0.986 |  | 0.032 (0.057) | 0.576 |  | 0.012 (0.083) | 0.882 |
| Model 2 | 0.007 (0.036) | 0.851 |  | 0.017 (0.047) | 0.711 |  | 0.020 (0.044) | 0.644 |  | 0.032 (0.056) | 0.575 |  | -0.004 (0.056) | 0.943 |  | -0.038 (0.081) | 0.640 |
| Linear regression, data is given as β (standard error). Model 1: crude model. Model 2: as model 1 plus adjustment for age, gender, sunbed use and long-lived family membership. Z-scores were used of CD45RA- CCR7- CD28- CD27-, CD57+ and KLRG1+ for normal distribution of data. | | | | | | | | | | | | | | | | | |

| **Supplementary table 4. P16INK4a positivity dependent on cytokine response upon flu peptide stimulation.** | | | | | | | | |  |
| --- | --- | --- | --- | --- | --- | --- | --- | --- | --- |
|  | CD4^+^ | | | |  | CD8^+^ | | | |
|  | Mean responders (N=51) | Mean non-responders (N=15) |  |  |  | Mean responders (N=36) | Mean non-responders (N=30) |  |  |
|  |  |  | OR (95% CI) | P-value |  |  |  | OR (95% CI) | P-value |
| Epidermal p16INK4a positivity |  |  |  |  |  |  |  |  |  |
| Model 1 | 1.911 | 0.907 | 1.173 (0.874; 1.573) | 0.288 |  | 1.683 | 1.682 | 1.000 (0.852; 1.174) | 0.999 |
| Model 2 | n/d | n/d | 1.276 (0.932; 1.746) | 0.128 |  | n/d | n/d | 1.015 (0.843; 1.223) | 0.871 |
| Model 3 | n/d | n/d | 1.275 (0.926; 1.756) | 0.137 |  | n/d | n/d | 1.009 (0.838; 1.215) | 0.923 |
|  |  |  |  |  |  |  |  |  |  |
| Dermal p16INK4a positivity |  |  |  |  |  |  |  |  |  |
| Model 1 | 2.449 | 1.645 | 1.187 (0.878; 1.605) | 0.265 |  | 2.601 | 1.865 | 1.146 (0.919; 1.429) | 0.225 |
| Model 2 | n/d | n/d | 1.093 (0.802; 1.490) | 0.573 |  | n/d | n/d | 1.138 (0.898; 1.442) | 0.284 |
| Model 3 | n/d | n/d | 1.084 (0.793; 1.480) | 0.613 |  | n/d | n/d | 1.142 (0.898; 1.453) | 0.279 |
| Responder: the percentage of cells producing IFNγ, TNF or IL-2 upon flu-peptide stimulation was ≥2x that in unstimulated cells. Logistic regression with 0=non-responder, 1=responder. Model 1 crude model, model 2 adjustment for age, gender, sunbed use and long-lived family membership, model 3 as model 2 plus adjustment for CMV serostatus. OR= odds ratio, CI = confidence interval, n/d = not done. | | | | | | | | | |
